# A Uniform for Narrating the Nurse in the Wheel of Time: An Interpretative Phenomenological Study

**DOI:** 10.1002/nop2.70166

**Published:** 2025-02-26

**Authors:** Jacopo Fiorini, Anna Marchetti, Angela Infante, Michela Piredda, Alessandro Sili

**Affiliations:** ^1^ Department of Nursing Professions University Hospital of Tor Vergata Rome Italy; ^2^ Department of Medicine and Surgery, Research Unit Nursing Science Università Campus Bio‐Medico di Roma Roma Italy; ^3^ Fondazione Policlinico Universitario Campus Bio‐Medico Rome Italy; ^4^ Department of Health Management University Hospital of Tor Vergata Rome Italy

**Keywords:** nurses, personal identity, professional role, social identification, uniforms

## Abstract

**Aim:**

To explore the symbolic and functional value that nurses attribute to their uniforms and profession.

**Design:**

An interpretative phenomenological study.

**Method:**

A purposeful sample of 15 nurses who designed their uniforms was enrolled between October 2022 and June 2023. A focus group was held to explore the participant‐designed uniforms, and an ideographic analysis was conducted.

**Results:**

Three main Experiential Statements emerged: nurses see their uniforms as tin armour, a wheel of time, and changes in time. Their professional and personal identities are shaped by their work, which also affects their time perception through past, present, and future perceptions. Nurses feel professionally motivated, even outside work, and despite society's recognition, uniforms symbolise their commitment. Nurses' identities are fused by the caring value, symbolised by their uniforms' evolution. Fostering self‐worth and professional values among nurses may inspire them, reduce attrition, and boost their professional recognition.

**Implications for Research and Practice:**

The study emphasises that nurses embrace dual identities as both professionals and citizens, which are unified by their commitment to patient care. Even outside of work, nurses still identify with their profession. Recognising and valuing the role of nurses may encourage more students to pursue nursing and reduce turnover in the profession. Healthcare organisations should implement strategies that underscore the importance of nurses in patient care and how their professional and personal identities shape their lives.

**Reporting Method:**

The COnsolidated criteria for REporting Qualitative research checklist was used to conduct and report this study.

**Patient or Public Contribution:**

No Patient or Public Contribution.

## 1 Introduction

In complex healthcare settings, nurses are usually the first professionals with whom patients interact when they enter a hospital and contribute significantly to ensuring patients' well‐being and satisfaction (Edvardsson et al. 2017). Since the birth of the nursing profession, nurses, especially female nurses, have been identified with caring for the fragile by their distinctive uniforms (Fitzgerald 2020; ten Hoeve et al. 2014).

The nurse's uniform is a recognisable icon of the healthcare field, symbolising professionalism, dedication and care toward patients (Hayward and Tuckey 2011). Over the years, the design of the nurse's uniform has evolved in tandem with the profession itself. The wearing of a uniform serves a dual purpose: to readily identify the individual as a nurse and to maintain hygienic standards. The nursing profession arose in its early days as a religious approach to caring for people in need, and therefore the first uniforms took on certain characteristics of this approach. For example, Dolan et al. (1978) described a historical nurse's uniform as ‘a plain blue cotton dress, with a white apron, a large turned down collar and a white muslin cap with a frill around the face, tied with a large bow beneath the chin of the wearer’. The evolution of the nurse's uniform reflects not only changes in societal norms and cultural contexts, but also advancements in textile technology and our understanding of hygiene. Nurses' attire has transitioned from informal and self‐made to standardised, functional, and professional garments (Hayward and Tuckey 2011), adapting to the practical, hygienic, and representative needs of the nursing profession (Fitzgerald 2020). From both an aesthetic and functional perspective, the nurse's uniform has undergone considerable changes in recent decades, adapting to the evolving demands of the healthcare sector while also reflecting a growing emphasis on the importance of patient comfort and well‐being (National Health System (NHS) England and NHS Improvement 2020). The uniform, beyond its symbolic role, allows nurses to be easily identified in the complex hospital environment and helps maintain proper hygiene standards. Moreover, the uniform reflects the culture and values of the nursing profession and has an impact on the psychological and emotional experience of both nurses and patients (Hayward and Tuckey 2011; Ke et al. 2017).

The nurse's uniform must meet mandatory requirements, although organisations may customise it. Common requirements for a nurse's hospital uniform are: comfort and a good fit, avoiding tight‐fitting or oversized garments, to guarantee adequate freedom of movement and minimise encumbrance; breathability, to allow for perspiration and cooling, ensuring comfort while working; ease of cleaning, with easily washable and easily sanitised materials, ensuring maximum hygiene and compliance with hospital standards; safety and fire resistance, without accessories or details that could expose patients or nurses themselves to risks; large and well‐positioned functional pockets, to allow nurses to carry and have fast and easy access to work tools and materials; professional aesthetics, to give nurses a professional and elegant look; protective against pathogens, creating a barrier between the nurse and the patient (National Health System (NHS) England and NHS Improvement 2020). Finally, beyond these fundamental requirements, individual healthcare organisations may add their distinctive custom elements.

The contemporary nurse's uniform is therefore the result of reflection on a combination of comfort, practicality and professional dignity (Hayward and Tuckey 2011). The nurse's uniform is a symbol of the profession. It identifies the nurse, strengthens team cohesion (O'Malley et al. 2024), and represents the nurse's commitment to providing quality care (Pearson et al. 2001). Donning the uniform, therefore, goes beyond simply putting on functional clothing. Indeed, Pearson et al. (2001) found that nurses perceived changing out of their everyday clothes into the uniform as a rite of passage, transforming them from layperson to healthcare professional. Changing clothes thus allows nurses to put aside their personal and family lives to fully enter the role of the professional, affirming their identity inside a healthcare organisation (Willetts and Clarke 2014). With constant commitment and dedication, their skills and abilities are then made available to patients, who recognise that nurses enter their lives to share emotions and make a difference in terms of their health and responding to their healthcare needs (Hayward and Tuckey 2011). Hence, the uniform represents a tangible symbol of recognition, responsibility, and professionalism, contributing to creating a sense of cohesion and belonging to one's professional community, and sharing values and objectives (Adamy et al. 2020).

To date, only two qualitative studies in the sector literature have investigated the nurse's uniform as a symbol of professional identification and testimony of belonging to the professional community (Hayward and Tuckey 2011; Pearson et al. 2001; Rasmussen et al. 2018). However, these studies were not aimed at exploring how the uniform represents aspects of personal identity.

This research constructs a narrative of the professional and individual lives of nurses wearing their uniforms through words and art. The aim is to explore the symbolic and practical value that nurses attribute to their uniforms and profession, examining what it means for nurses to wear their uniforms by fusing the story of their experiences with the material representation of their image as individuals and health professionals.

## 2 Methods

An interpretive phenomenological (IPA) study was conducted. IPA was used for exploring personal lived experiences, the meaning of these experiences and how people embody those (Larkin et al. 2021). This approach was integrated by the use of art, according to the ideographic principles (Smith 1996) and the performative perspective (Bode 2010). Given the study aimed to explore the symbolic and functional value that nurses attribute to their uniform and their profession, IPA aligned with art was an adaptable and accessible approach achieving a complete and in‐depth interpretation of these values. The COnsolidated Criteria for REporting Qualitative Research (COREQ) checklist was followed for the preparation and conduct of the study (Tong et al. 2007).

### 2.1 Sample

Between October 2022 and June 2023, a purposeful sample of nurses was recruited for the study to ensure a heterogeneous representation based on age, gender, family status, clinical area (e.g., medical, surgical, intensive care), and work experience (e.g., hospital, community). This approach was chosen to capture different perspectives on nurses' uniforms and identity (Bradshaw et al. 2017; Sandelowski 2000). Registered nurses were eligible to participate if they had at least 1 year of work experience, wore a uniform at work, and were willing to provide informed consent to participate. Midwives, nursing directors, assistants, and students were excluded. Potential participants who met the eligibility criteria were contacted via email and invited to participate. The email provided details about the study and requested their consent. Due to the study design and methodology, a small sample, ranging from 4 to 20, is considered appropriate to explore the nurses' uniforms and professional values (Larkin et al. 2021).

### 2.2 Data Collection

The data collection was divided into three sessions. In the first two‐hour session, participants were provided with creative craft materials, including colouring materials, glitter and sheets of paper, as well as a laminated card showing the following questions: ‘What does being a nurse mean to you?’, and ‘What does the uniform you wear every day represent for you?’. They were asked to answer these questions ideographically and individually, representing their personalised nurse's uniform on paper. In the second session, a 1‐h focus group was conducted involving all the participants (*n* = 15), and the discussion was audio‐recorded and transcribed verbatim. The moderator and observers of the focus group were professionals with a PhD and experienced in using this research technique, and external to the nurses' organisation, to avoid any undue influence on the participants. The focus group began by asking the participants what they had depicted on the sheets of paper and its meaning. Instead of individual interviews, researchers used focus groups to stimulate participants to personally articulate what they wanted to show and the meaning behind their nurses' uniform representations. This methodological approach also facilitated the collective exploration of uniform symbols participants shared openly and collaboratively their emotions, ideas and beliefs, thus integrating latent individual and collective meanings (Nyumba et al. 2018). Data saturation was reached when the thematic discussion became homogeneous and redundant and participants agreed with the uniform symbolism meanings (Hennink et al. 2019). In‐depth data was therefore obtained from each participant, as demanded by the performative perspective (Bode 2010). In the third session, the participants were provided with white uniforms and various materials, such as pieces of cloth, paints, and shoes, to realise in 4 h the uniform they had designed in the first session, and sociodemographic (age, gender) and working variables (length of service, clinical work area) were collected. The participants' privacy was guaranteed so that in the sessions they felt free to communicate their emotions, ideas and beliefs without any filtering or fear of retaliation.

### 2.3 Data Extraction and Synthesis

After the conclusion of the focus group, the research team held a debriefing to share impressions of how the group discussion went, the topics discussed, and the characteristics of the group. Collected words, emotions, and notes were then transcribed. Following (Larkin et al. 2021; Smith 1996), the idiographic analysis of the material created was incorporated into a thematic analysis. The transcripts were read several times to deeply explore the symbolic and functional value of uniforms and the nursing profession for participants. After this reading, the research team performed a free text analysis to identify meaning units. In this stage, the data was compared, searching for associations and similarities, and was linguistically reformulated in annotations. Subsequently, an emerging thematic analysis was performed at the abstraction level, invoking free imaginative concepts with a focus on the transcription, the annotations, and the idiographic representations.

The themes identified were listed and organised into clusters of Experiential Statements and Personal Experiential Themes, categorised according to their prevalence within the data, the depth and symbolic value of their meanings, and their relationship with other themes. The analysis then used iterative free imaginative variation to consider different Experiential Statements and their constant and essential elements concerning the words expressed by the participants and the realised uniforms. Through this process, a general structure and essential elements were identified for describing the nurses' experiences of wearing their uniforms and their perspectives on their professional and personal identities (Tong et al. 2007). Finally, the sociodemographic and organisational variables were analysed with a descriptive approach using the Statistical Package for the Social Sciences v.22, considering measures of percentile, mean, standard deviation, minimum, and maximum.

### 2.4 Rigour

To ensure the rigour of the study, the researchers followed the principles of qualitative research (Lincoln and Guba 1994; Sferra 2005). The following criteria have been respected: credibility (with certain strategies to ensure the credibility of the results), reliability (with the use of approaches designed for replicability of the results), confirmability (by guaranteeing a faithful representation of the participants' narratives and asking their feedback after the findings' writing), transferability (by considering how the study's findings fit other contexts similar to the study area), authenticity (by providing details of participants' experiences and feelings concerning the studied phenomenon) and reflection (by continuous questioning stance to maintain research team focused and analysing by seeking convergence, divergence and representativeness) (Polit and Beck 2008). To manage reflexivity, during the data collection and analysis, the research team documented methodological choices, individual perceptions, potential bias, group interactions, and coding frameworks in journal notes. An audit of the process was undertaken from initial notes to the final draft to determine whether an independent auditor could follow the steps (Larkin et al. 2021).

These criteria were guaranteed in the study through the detailed drafting of the method, the enrolment of participants with heterogeneous characteristics, and the granting of freedom to realise their uniforms using any available material. This freedom respected their performative perspective regarding the uniforms and the professional values of nursing (Bode 2010). Finally, only the units of meaning that appeared with a reasonable frequency in the focus group and the symbolic values most identified in the idiographic and material realisation of the uniforms were included in the data analysis.

### 2.5 Ethical Considerations

The study was approved in advance by the Ethics Committee of the hospital where it was conducted (RS. Prot. 747/23) by the principles of the Declaration of Helsinki (World Medical Association 2024). Each potential participant received detailed information about the study, including the opportunity to discuss it with the researchers and to reflect on their participation. Written informed consent was issued before proceeding with the data collection. All data was treated with confidentiality and kept in a secure location with limited access.

## 3 Results

In the study, 15 nurses were enrolled, of which 60% (*N* = 9) were women, with an average age of 50.97 years (SD = 8.42; range 31–68) and a length of service of 19.34 years (SD = 3.82; range 7–23). Concerning the clinical work area, 33.4% (*N* = 5) worked in surgery, 20% (*N* = 3) worked in neurology, and the remainder worked respectively in medicine (*N* = 2; 13.3%), emergency (*N* = 2; 13.3%), cardiology (*N* = 2; 13.3%), and haematology (*N* = 1; 6.70%).

### 3.1 General Structure: The Wheel of Time

The general structure emerging from the results was the wheel of time, that is, a symbol consisting of a motionless and immutable circular form that generates movement and transformation, with spokes that support the entire structure, are oriented in different directions, and testify to renewal and continuous regeneration. The wheel of time metaphorically represents nurses with their uniforms, who, in time, are transformed in a world that changes and faces health challenges that inevitably affect the nurses' personal and professional lives, although they continue to assist patients and support the healthcare system. The general structure is based on three Experiential Statements, namely professional identity in time, personal identity in time, and the identity of nurses in contemporary society. Each Experiential Statement described in the following paragraphs has at least one Personal Experiential Theme and brings together the words of the participants and their representations of their uniforms. The Experiential Statements, Personal Experiential Themes and illustrative quotes have been summarised in Table 1.

**TABLE 1:** Experiential statements, personal experiential themes, and illustrative quotes.

Experiential statements	Personal experiential themes	Illustrative quotes
Professional identity in time	*The nurse before and now*	‘I would like you to accompany me inside my uniform, it will tell you about my journey of being a nurse’
		‘Looking at myself in the mirror wearing my uniform, I asked myself, ‘Who is this nurse?’ Someone, no‐one, one of a hundred thousand?’ (Figure 1)
	*The tin armour I wear every day*	‘My uniform is like tin armour, it defends me and identifies me as a health professional’
		‘We must first take care of ourselves and our families, and then we will be able to meet the others in their moment of need, with our words, gestures and silences’ (Figure 2)
Personal identity in time	*Perception of time: Chronos, Aion, Kairos*	‘My life as a nurse is like a prisoner sun, unique and powerful, embracing everything, but clouds can obscure it, and make it difficult to give energy and warmth to one's fellow human beings’.
		‘I have experienced a journey just like the uniform I wear, within myself, between desires and aspirations, discovering strengths and weaknesses’ (Figure 2)
The identity of the nurse in contemporary society	*The lack of recognition of nurses today*	‘We felt like heroes in a parenthesis of life that opened up a dark moment of humanity. Then it all ended and what about us? We were damaged, destroyed, tired, poor, worn out… nurses in the trenches helping out all those who extended their hand seeking it’ (Figure 3)
	*How to be a nurse: a tree in a forest*	‘I represent being a nurse through my uniform, like the bark of a tree, with roots penetrating the health system, and a trunk that allows me to support the organisation, spreading sap, even in the driest terrain’(Figure 1)

### 3.2 Professional Identity in Time

**FIGURE 1:**
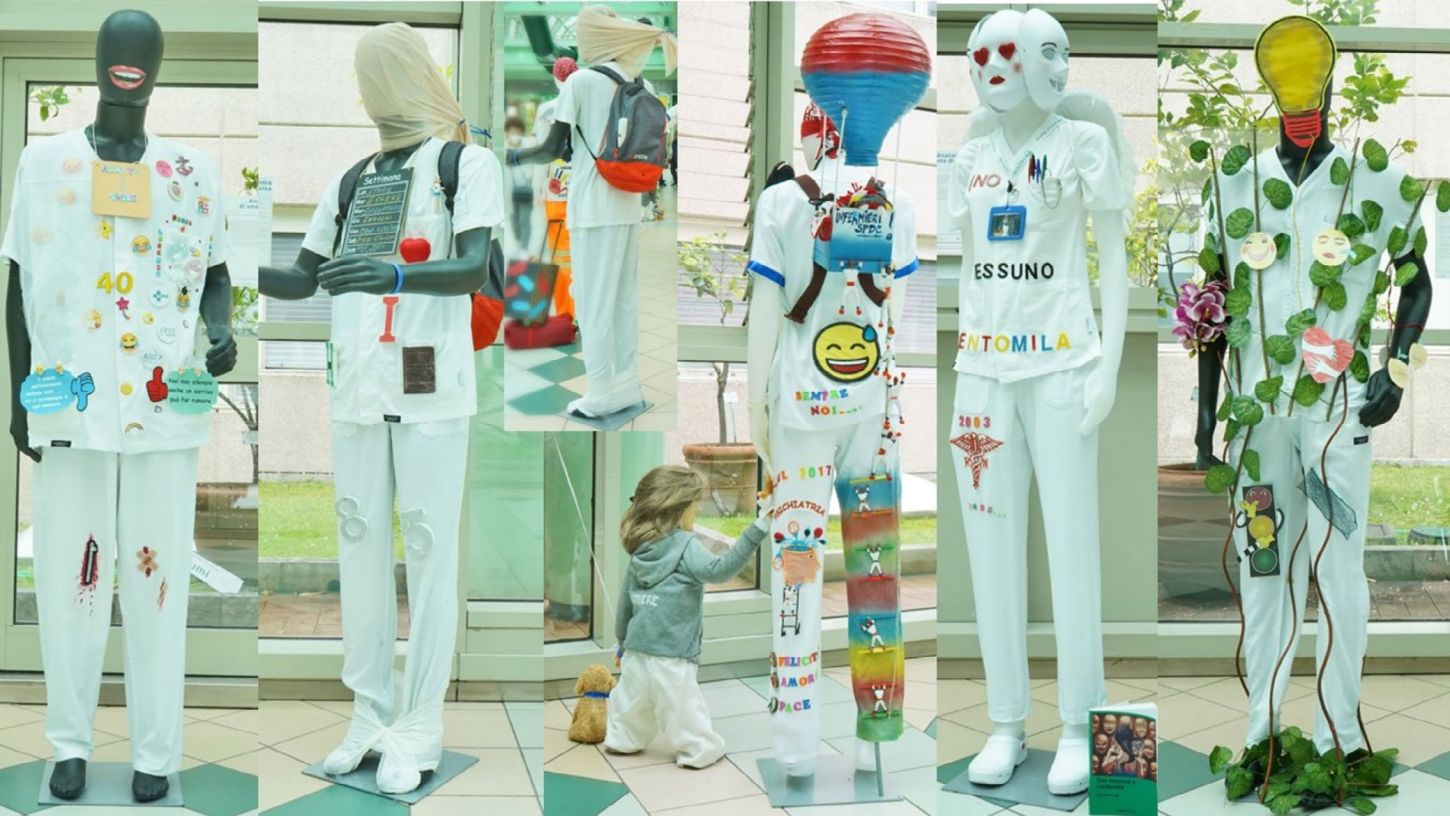
The uniforms created by the participants represent the nursing profession as a journey, highlighting the multifaceted roles of nurses as both individuals and professionals.

*The nurse before and now—*The nurses described their uniform as a symbol of their professional identity, of their role in the healthcare sector, and of the emotions they feel every day, with such words as ‘I would like you to accompany me inside my uniform, it will tell you about my journey of being a nurse’. They told of their professional identity changing over the years—‘Our history is sewn onto our uniforms’—and of the value they attributed to the uniform they wear every day—‘Looking at myself in the mirror wearing my uniform, I asked myself, ‘Who is this nurse?’ Someone, no‐one, one of a hundred thousand?’ (Figure 1). The nurses previously felt they were seen as ‘supportive of other healthcare professions, without having decision‐making autonomy’. Today, for them, being a nurse meant being a graduated healthcare professional, responsible for patient care and organisational practices—‘We know that when you are sick, you can't enter a hospital and not see a doctor. We are surprised to learn that patients cannot leave a hospital in a better condition than when they entered without a nurse’, being a superhero ‘with a capital S’ in the healthcare setting, and being close to patients 24 h a day, 365 days a year.

**FIGURE 2:**
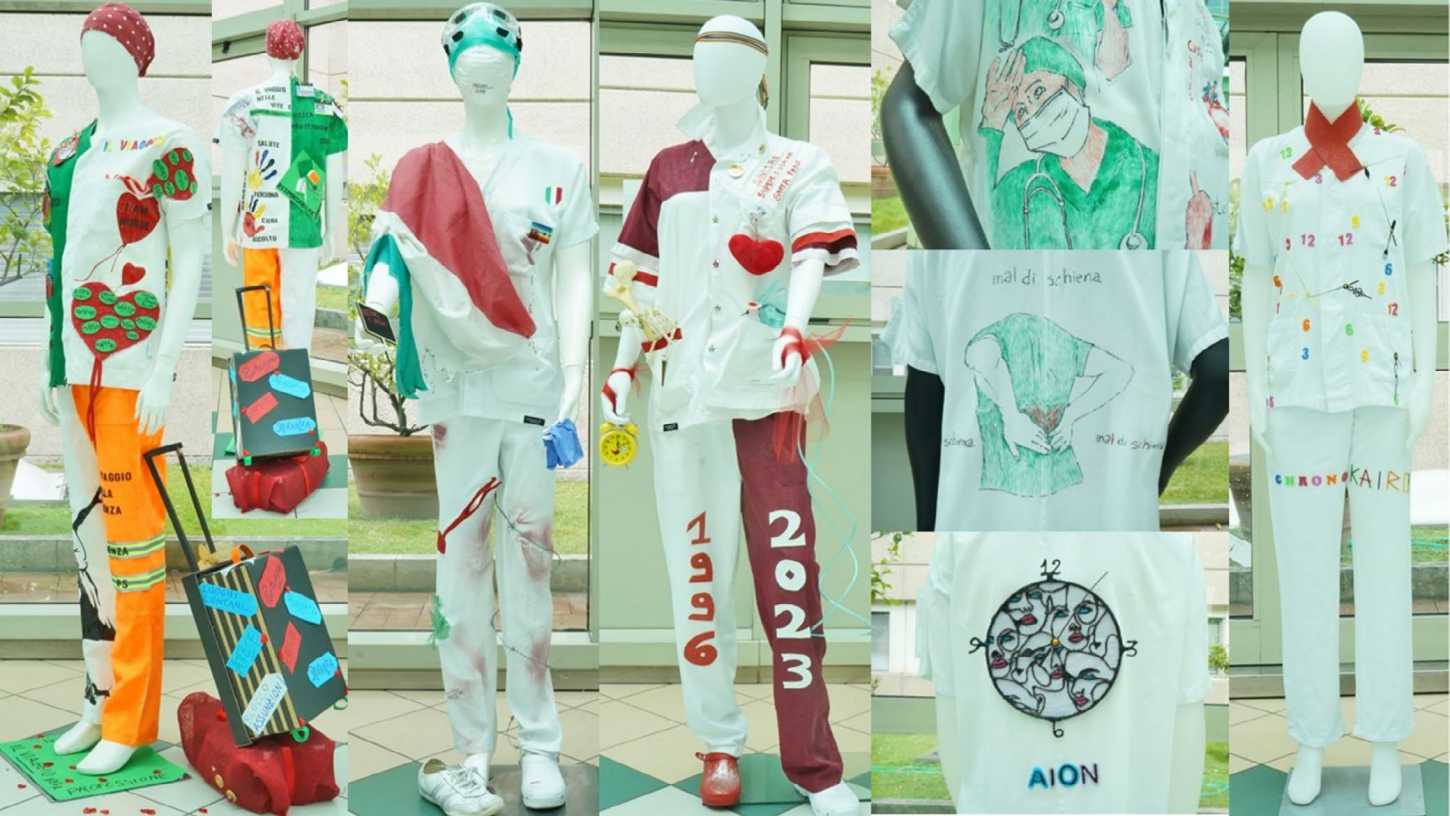
The uniforms created by the participants symbolise the passage of time and honour national heroes.

*The tin armour I wear every day—*The uniform that nurses wore every day was capable of telling their stories and experiences in their clinical area and the care of their patients, as the nurses shared within their discussions—‘I hide my tears and bring smiles to the faces of the people I help’, ‘My uniform is like tin armour, it defends me and identifies me as a health professional’. The participants reported that wearing their uniforms allowed them to embrace the healthcare setting, by undressing out of their personal lives to enter their healthcare lives (Figure 2). This armour was not considered to be merely physical, but also a shield of emotional resilience and professional identity—‘I touch the soul of my patients with a smile because a smile dispels doubts and brings comfort’, ‘My hands welcome, support, accompany, act, touch, revive and greet life’. They felt they were able to live in the present and manage the balance between work and family, acting with awareness to seize the critical moment, *Kairos*—‘We must first take care of ourselves and our families, and then we will be able to meet the others in their moment of need, with our words, gestures and silences’ (Figure 2). This approach allowed the nurses to give meaning to what happens in life and to look at things from a different perspective, from which ‘Nurses bring care and calmness to an environment full of emotion, suffering and pain’.

### 3.3 Personal Identity in Time

Participants also reported how their professional development involved personal growth. As represented by some uniforms, participants distinguished ways of perceiving their time as: (Figure 2) quantitative time, *Chronos*; perpetual time, *Aion*; and the quality of time, *Kairos*. These three concepts represented different ways of interpreting time and its influence on their daily lives. For example, *Chronos* was referenced as ‘A giant who devours time’. Furthermore, the frenetic rhythms of work could overwhelm nurses on a personal level, oppressing them, suffocating them, or binding them in chains—‘My life as a nurse is like a prisoner sun, unique and powerful, embracing everything, but clouds can obscure it, and make it difficult to give energy and warmth to one's fellow human beings’. This *Aionic* metaphorical darkening, representing a limitation of their personal freedom, prevented them from developing curiosity or living life with serenity. Finally, *Kairos* was represented as ‘a young man in constant movement’, symbolising the right moment to live and act—‘I have experienced a journey just like the uniform I wear, within myself, between desires and aspirations, discovering strengths and weaknesses’. This sense of time invited the nurses to live in the present with awareness, ‘letting go of what limits me, like a balloon that flies away, and jealously holding onto everything that enriches me’.

### 3.4 The Identity of the Nurse in Contemporary Society

**FIGURE 3:**
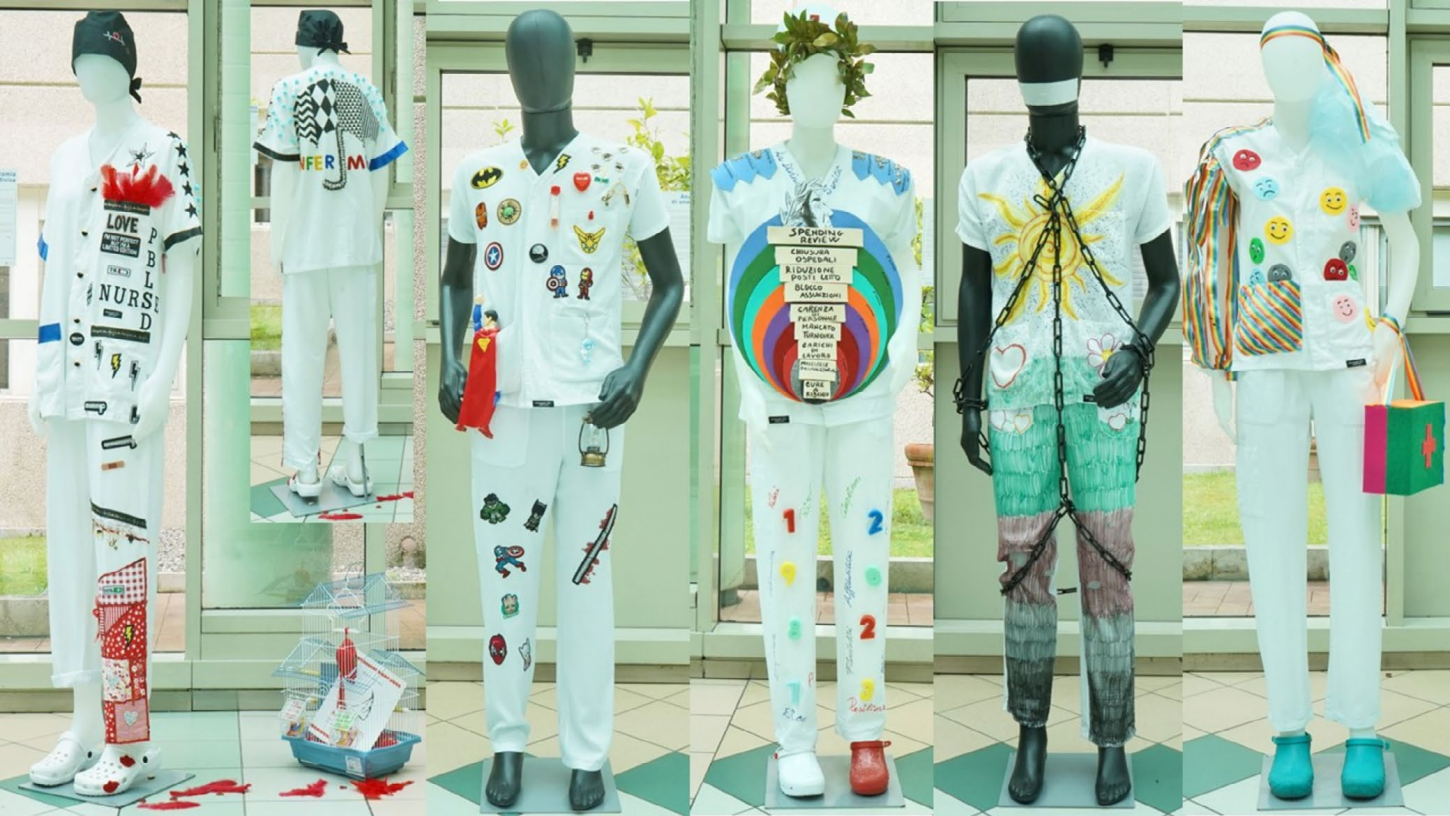
The uniforms created by the participants reflect the perceived lack of recognition from the public after the pandemic, as well as the emotions experienced in their clinical practice.

*The lack of recognition of nurses today—*This theme reflects the perception that the participants reported of their profession from the society in which they live. The public perception of nurses and their importance in society was often felt to be taken for granted (Figure 3)—‘We are often presented with obstacles on our way to social and work recognition’. Even though the nurses felt that their skills were not fully recognised or understood by citizens and politicians, they suggested that they are in an ‘infernal circle of a collapsing healthcare system, overwhelmed by increasingly demanding challenges, spending reviews, lack of turnover, and abandonment of the profession’. This negative perception by society was discerned despite the crucial role they felt they played in healthcare, particularly during the COVID‐19 pandemic—‘We felt like heroes in a parenthesis of life that opened up a dark moment of humanity. Then it all ended and what about us? We were damaged, destroyed, tired, poor, worn out… nurses in the trenches helping out all those who extended their hand seeking it’.

*How to be a nurse: a tree in a forest—*Despite the perceived public opinion, the participants expressed that resilience and passion for their profession were the aspects that motivated them to continually face daily challenges and difficulties—‘I feel emotions, cry tears of pain and joy, but, despite everything, I believe in my profession: a modern nurse, a professional with advanced skills’, ‘I dreamed of becoming a nurse since I was a child, and today I am a nurse’ (Figure 1). Regardless of the clinical work area, all the participants expressed their commitment to guaranteeing high‐quality care. Despite the demanding working conditions and the lack of adequate social recognition, ‘I represent being a nurse through my uniform, like the bark of a tree, with roots penetrating the health system, and a trunk that allows me to support the organisation, spreading sap, even in the driest terrain… We always find alternative ways to get through to our patients… as the branches of a tree orient themselves towards the sun’.

## 4 Discussion

This study investigated the symbolic and functional value that nurses attribute to their uniforms and their profession. The results provide a broad perspective on the perception of nurses of their identity as individuals and as healthcare professionals in society and in time. It reveals a strong professional identity and dedication to patient care, in both work‐related situations, such as during the pandemic, and personal aspects, where family issues are set aside while wearing the uniform. The nurse uniform is symbolised by a wheel, as an unchanging and unifying structure, with spokes oriented in all directions and allowing movement and action, signifying how a nurse, as a person and a professional, is an integral part of society and evolves to face emerging healthcare challenges while maintaining a professional commitment to patient care (ten Hoeve et al. 2014).

When nurses arrive at work, they shed their everyday clothes, representing their personal and family lives, to don their uniforms. With a smile, a touch, and carefully judged silence, they approach patients to fulfil their needs (Pearson et al. 2001). The uniform is a kind of tin armour to be worn and identified as a health professional, to serve citizens, and to be appreciated by the public. Without the uniform, other professionals and citizens would not be able to identify nurses (Hayward and Tuckey 2011). Wearing it grants them the symbolic title of a superhero. Such findings align with previous phenomenological research, which has identified wearing the uniform as a rite of passage (Ke et al. 2017; Pearson et al. 2001). The various aspects of a nurse's identity highlight the coexistence of personal and professional dimensions that shape the healthcare professionals (Fitzgerald 2020; ten Hoeve et al. 2014; Rasmussen et al. 2018; Schmidt and McArthur 2018).

Recent investigations into professional identity have highlighted its foundation in values, beliefs, and ethics, in context and socialisation, and group and social identities (Adamy et al. 2020; Andrew 2012; Fitzgerald 2020). Studies on nurses' quality of life underscore the importance of understanding both personal and professional identities to ensure nurses' well‐being at work and affect job satisfaction, the sense of belonging to one's organisation, the quality of the performance of one's work, and finally the quality of care perceived by patients (Prottas and Nummelin 2018; Sabancıogullari and Dogan 2015; Sili et al. 2022).

The representation of the uniform as tin armour evokes the battle of nurses and healthcare professionals against the COVID‐19 pandemic. Our findings suggest that the public acclaim of nurses as superheroes was transient, closely linked to the pandemic, and has not been sustained over time. Nurses do not feel recognised for the role they play in healthcare organisations daily, despite, for instance, being the first to meet patients when they arrive at the emergency department, or being constantly present in wards (Firew et al. 2020). This lack of recognition was also represented in a uniform with a double‐sided clock, testifying to time spent and involvement in patient care. While the dedication of nurses has always been evident, the COVID‐19 pandemic brought to light the true value and role of these professionals in healthcare (Ulrich et al. 2020; Zaghini et al. 2021). One participant in this study even decided to associate the uniform with the national flag, as a symbol of national pride. Nurses, of course, experienced the pandemic as both healthcare professionals and citizens, taking on one role or the other when they tested positive for COVID‐19 or became aware of their condition or the risks inherent in their work for both themselves and the family members they lived with (Piredda et al. 2022). They also suffered the stigma of being a possible source of virus transmission (Fernandez et al. 2020). Society's perception of nurses influences their lives and their professional identity. While acknowledgment brings gratification, the lack of it engenders hurt. Despite this, every day, nurses continue their work with dedication, and having contracted the virus brought them closer to those care practices that they previously took for granted, such as simple physical contact and the shaking of hands with a patient (Piredda et al. 2022). Various footwear types worn by mannequins and chains encircling the uniforms depicted the disappointment of the lack of recognition at the end of the pandemic in this study.

The participants also emphasised that being a nurse transcended wearing a uniform. Even after removing their work attire and returning to their everyday casual clothes, they brought with them the emotions and experiences shared with their patients. What nurses experience during their work shifts is thus packed into and becomes part of their emotional baggage (Hayward and Tuckey 2011; Pearson et al. 2001), which, as represented by a mannequin directed by one participant of this study, is carried by each nurse on his/her life's journey, and which characterises and differentiates him/her from others. Whether or not a nurse is wearing his/her uniform, he/she remains a nurse even after working hours. This sense of affirmation allows us to bring together the division proposed by Pearson et al. (2001) between family and work life and explains the results of multiple quantitative studies that have highlighted the relationship between work–family conflict and the quality of care perceived by patients. Simply put, a nurse who successfully balances work and family demands delivers better job performance, is less stressed, has less risk of developing burnout, and improves the quality of the care provided (Mauno et al. 2012; Molina 2021).

Work rhythms often imprison nurses in repetitive actions, and monotony can lead them to detach themselves from the care actions they perform (Bakker and Heuven 2006). This disengagement is also a defence mechanism that they can sometimes use to avoid getting involved in the emotions of their clients or in challenging clinical and organisational situations that might demand greater commitment and involvement (Zaghini et al. 2020; Zapf and Holz 2006). They therefore try to limit their emotional involvement to avoid developing exhaustion and burnout (Sili et al. 2014). The nursing profession, however, is fundamentally based on the relationship with the person being cared for, and the participants of this study, despite recognising the onus of responsibility, felt they dedicated their time, in terms of both quality and quantity, to their patients without feeling it as a real burden (Zerbini et al. 2020). The motivation still felt by participants—who had been working on average for 20 years in clinical settings—which drove them to become nurses and care for patients, was an essential aspect that emerged from this study, confirming previous research on the professional identity of nurses (Fitzgerald 2020; ten Hoeve et al. 2014). It is perhaps surprising that the participants still so keenly expressed this motivational aspect after so many years. They talked about their dedication to the profession and the recognition of their work in this historical moment in which the rate of abandonment of the nursing profession is increasing, and the number of nurses is far below the standard needed to maintain a good quality of care (van der Cingel and Brouwer 2021). It would be interesting to investigate, through future qualitative studies, this motivation since the perspective may be useful for encouraging nurses wanting to leave the profession to stay and for encouraging nursing students to approach and enter the profession with the same determination and to maintain it constantly over the years of their careers.

### 4.1 Strengths and Limitations

A rigorous methodology is one strength of this study, especially in its execution and analysis of the collected data. However, the findings must be interpreted while taking account of certain limitations. The qualitative study was conducted in a single country (Italy), within one hospital, and involved a small sample, which limits the representativeness of how all nurses perceive their identities and uniforms. Nonetheless, the participants had heterogeneous work experiences in a variety of clinical settings.

### 4.2 Implications for Research and Practice

Becoming a nurse in this century is a challenge, and patient care motivation is key to preventing abandonment of the profession. This study highlights nurses' dual identities as professionals and citizens brought together in recognising the value of caring for patients. The nurse's uniform represents the moment of being on duty and responding to patients' needs. However, even when they wear their out‐of‐work clothing, they remain and still identify as nurses.

Identifying motivation and promoting recognition of the value of being a nurse may stimulate students to enrol in university nursing courses and help stem nurses' intention to leave the profession. Healthcare organisations should therefore implement interventions such as stimulating professional identity growth or establishing recognition and appreciation feedback, to highlight the role of nurses in the patient care process (Willetts and Clarke 2014). These initiatives can contribute to shaping nurses' professional and personal identities and define their lives, thus improving caring and organisational success.

## 5 Conclusion

This study investigated the symbolic and functional value that nurses attribute to their uniforms and profession, capturing some of the essence of the nursing profession by examining their feelings about their uniforms and thereby shedding light on nurses' perception of time, professional identity and the humanisation of care as key factors that influence nurses' job satisfaction and retention. Their perception of time reflects a delicate balance between individual and professional responsibilities, while the professional identity of a nurse serves as a protective armour and a source of resilience. Moreover, the humanisation of care highlights the relationship established by nurses with their patients, which promotes their sense of purpose and fulfilment. Lastly, social recognition of nurses and their profession reinforces their importance in society. To advance the nursing profession, promoting the identity of nurses, improving their working conditions, and raising awareness of their crucial role in contemporary society are essential to attracting and retaining professionals in the healthcare sector.

## Author Contributions

Jacopo Fiorini: conceptualization, methodology, investigation, formal analysis, data curation, visualisation, writing – original draft, and writing – review and editing. Michela Piredda and Anna Marchetti: methodology, visualisation, and writing – original draft. Angela Infante: conceptualization, investigation and data curation. Alessandro Sili: conceptualization, methodology, writing – review and editing, and supervision. All authors contributed to the article and approved the submitted version.

## Conflicts of Interest

The authors declare no conflicts of interest.

## Data Availability

The data that support the findings of this study are available from the corresponding author upon reasonable request.

## References

[nop270166-bib-0001] Adamy, E. K. , D. A. d. A. Zocche , and M. d. A. Almeida . 2020. “Contribution of the Nursing Process for the Construction of the Identity of Nursing Professionals.” Revista Gaúcha de Enfermagem 41, no. spe: 1–8. 10.1590/1983-1447.2020.20190143. 31778384

[nop270166-bib-0002] Andrew, N. 2012. “Professional Identity in Nursing: Are We There Yet?” Nurse Education Today 32, no. 8: 846–849. 10.1016/j.nedt.2012.03.014. 22531469

[nop270166-bib-0003] Bakker, A. B. , and E. Heuven . 2006. “Emotional Dissonance, Burnout, and In‐Role Performance Among Nurses and Police Officers.” International Journal of Stress Management 13, no. 4: 423–440. 10.1037/1072-5245.13.4.423.

[nop270166-bib-0004] Bode, M. 2010. “Showing Doing. The Art‐Science Debate in a Performative Perspective.” Journal of Consumer Behaviour 9, no. 2: 139–155. 10.1002/cb.310.

[nop270166-bib-0005] Bradshaw, C. , S. Atkinson , and O. Doody . 2017. “Employing a Qualitative Description Approach in Health Care Research.” Global Qualitative Nursing Research 4: 282. 10.1177/2333393617742282. PMC5703087 29204457

[nop270166-bib-0006] Dolan, J. A. , M. L. Fitzpatrick , and E. K. Herrmann . 1978. Nursing in Society: A Historical Perspective. Saunders.

[nop270166-bib-0007] Edvardsson, D. , E. Watt , and F. Pearce . 2017. “Patient Experiences of Caring and Person‐Centredness Are Associated With Perceived Nursing Care Quality.” Journal of Advanced Nursing 73, no. 1: 217–227. 10.1111/jan.13105. 27532230

[nop270166-bib-0008] Fernandez, R. , H. Lord , E. Halcomb , et al. 2020. “Implications for COVID‐19: A Systematic Review of Nurses' Experiences of Working in Acute Care Hospital Settings During a Respiratory Pandemic.” International Journal of Nursing Studies 111: 103637. 10.1016/j.ijnurstu.2020.103637. 32919358 PMC7206441

[nop270166-bib-0009] Firew, T. , E. D. Sano , J. W. Lee , et al. 2020. “Protecting the Front Line: A Cross‐Sectional Survey Analysis of the Occupational Factors Contributing to Healthcare Workers' Infection and Psychological Distress During the COVID‐19 Pandemic in the USA.” BMJ Open 10, no. 10: 1–12. 10.1136/bmjopen-2020-042752. PMC7580061 33087382

[nop270166-bib-0010] Fitzgerald, A. 2020. “Professional Identity: A Concept Analysis.” Nursing Forum 55, no. 3: 447–472. 10.1111/nuf.12450. 32249453

[nop270166-bib-0011] Hayward, R. M. , and M. R. Tuckey . 2011. “Emotions in Uniform: How Nurses Regulate Emotion at Work via Emotional Boundaries.” Human Relations 64, no. 11: 1501–1523. 10.1177/0018726711419539.

[nop270166-bib-0012] Hennink, M. M. , B. N. Kaiser , and M. B. Weber . 2019. “What Influences Saturation? Estimating Sample Sizes in Focus Group Research.” Qualitative Health Research 29, no. 10: 1483–1496. 10.1177/1049732318821692. 30628545 PMC6635912

[nop270166-bib-0013] Ke, Y. , C. Kuo , and C. Hung . 2017. “The Effects of Nursing Preceptorship on New Nurses' Competence, Professional Socialization, Job Satisfaction and Retention: A Systematic Review.” Journal of Advanced Nursing 73, no. 10: 2296–2305. 10.1111/jan.13317. 28398636

[nop270166-bib-0014] Larkin, M. , P. Flowers , J. A. A. Smith , and M. Osborne . 2021. Interpretative Phenomenological Analysis: Theory, Method and Research. SAGE Publications Ltd.

[nop270166-bib-0015] Lincoln, Y. S. , and E. G. Guba . 1994. “Paradigmatic Controversies, Contradictions and Emerging Confluences.” In Handbook of Qualitative Research, 2nd ed., 163–194. Sage Publications.

[nop270166-bib-0016] Mauno, S. , U. Kinnunen , J. Rantanen , T. Feldt , and M. Rantanen . 2012. “Relationships of Work—Family Coping Strategies With Work—Family Conflict and Enrichment.” Family Science 3, no. 2: 109–125.

[nop270166-bib-0017] Molina, J. A. 2021. “The Work‐Family Conflict: Evidence From the Recent Decade and Lines of Future Research.” Journal of Family and Economic Issues 42, no. S1: 4–10. 10.1007/s10834-020-09700-0.

[nop270166-bib-0018] National Health System (NHS) England , and NHS Improvement . 2020. “Uniforms and Workwear: Guidance on Uniform and Workwear Policies for NHS Employers.”

[nop270166-bib-0019] Nyumba, T. O. , K. Wilson , C. J. Derrick , and N. Mukherjee . 2018. “The Use of Focus Group Discussion Methodology: Insights From Two Decades of Application in Conservation.” Methods in Ecology and Evolution 9, no. 1: 20–32. 10.1111/2041-210X.12860.

[nop270166-bib-0020] O'Malley, M. , J. O'Mahony , B. Happell , and H. Mulcahy . 2024. “The Nurse Bombarded, Consumed and Vulnerable: An Interpretative Phenomenological Analysis of Mental Health Nurses' Self‐Care at Work.” Journal of Psychiatric and Mental Health Nursing 31, no. 1: 66–76. 10.1111/jpm.12956. 37534379

[nop270166-bib-0021] Pearson, A. , H. Baker , K. Walsh , and M. Fitzgerald . 2001. “Contemporary Nurses' Uniforms‐History and Traditions.” Journal of Nursing Management 9, no. 3: 147–152. 10.1046/j.1365-2834.2001.00207.x. 11879461

[nop270166-bib-0022] Piredda, M. , J. Fiorini , A. Marchetti , et al. 2022. “The Wounded Healer: A Phenomenological Study on Hospital Nurses Who Contracted COVID‐19.” Frontiers in Public Health 10: 1–9. 10.3389/fpubh.2022.867826. PMC9302606 35875015

[nop270166-bib-0023] Polit, D. F. , and C. T. Beck . 2008. Nursing Research: Generating and Assessing Evidence for Nursing Practice. 8th ed. Wolters Kluwer Health/Lippincott Williams & Wilkins.

[nop270166-bib-0024] Prottas, D. J. , and M. R. Nummelin . 2018. “Behavioral Integrity, Engagement, Organizational Citizenship Behavior, and Service Quality in a Healthcare Setting.” Journal of Healthcare Management 63, no. 6: 410–424. 10.1097/JHM-D-17-00134. 30418370

[nop270166-bib-0025] Rasmussen, P. , A. Henderson , N. Andrew , and T. Conroy . 2018. “Factors Influencing Registered Nurses' Perceptions of Their Professional Identity: An Integrative Literature Review.” Journal of Continuing Education in Nursing 49, no. 5: 225–232. 10.3928/00220124-20180417-08. 29701865

[nop270166-bib-0026] Sabancıogullari, S. , and S. Dogan . 2015. “Effects of the Professional Identity Development Programme on the Professional Identity, Job Satisfaction and Burnout Levels of Nurses: A Pilot Study.” International Journal of Nursing Practice 21, no. 6: 847–857. 10.1111/ijn.12330. 24779558

[nop270166-bib-0027] Sandelowski, M. 2000. “Focus on Research Methods: Whatever Happened to Qualitative Description?” Research in Nursing and Health 23, no. 4: 334–340. 10.1002/1098-240x(200008)23:4<334::aid-nur9>3.0.co;2-g. 10940958

[nop270166-bib-0028] Schmidt, B. J. , and E. C. McArthur . 2018. “Professional Nursing Values: A Concept Analysis.” Nursing Forum 53, no. 1: 69–75. 10.1111/nuf.12211. 29419942

[nop270166-bib-0029] Sferra, F. 2005. “Constructing the Wheel of Time. Strategies for Establishing a Tradition.” In Boundaries, Dynamics and Construction of Tradition in South Asia, edited by F. Squarcini , 253–285. Firenze University Press.

[nop270166-bib-0030] Sili, A. , M. De Maria , J. Fiorini , F. Zaghini , and C. Barbaranelli . 2022. “Nurses' Quality of Life Scale: Validation and Psychometric Properties.” Evaluation & the Health Professions 45, no. 3: 016327872210756. 10.1177/01632787221075660. 35081784

[nop270166-bib-0031] Sili, A. , R. Fida , F. Zaghini , C. Tramontano , and M. Paciello . 2014. “Counterproductive Behaviors and Moral Disengagement of Nurses as Potential Consequences of Stress‐Related Work: Validity and Reliability of Measurement Scales.” La Medicina del Lavoro 105, no. 5: 382–394. http://www.ncbi.nlm.nih.gov/pubmed/25134633. 25134633

[nop270166-bib-0032] Smith, J. A. 1996. “Beyond the Divide Between Cognition and Discourse: Using Interpretative Phenomenological Analysis in Health Psychology.” Psychology and Health 11, no. 2: 261–272.

[nop270166-bib-0033] ten Hoeve, Y. , G. Jansen , and P. Roodbol . 2014. “The Nursing Profession: Public Image, Self‐Concept and Professional Identity. A Discussion Paper.” Journal of Advanced Nursing 70, no. 2: 295–309. 10.1111/jan.12177. 23711235

[nop270166-bib-0034] Tong, A. , P. Sainsbury , and J. Craig . 2007. “Consolidated Criteria for Reporting Qualitative Research (COREQ): A 32‐Item Checklist for Interviews and Focus Groups.” International Journal for Quality in Health Care 19, no. 6: 349–357. 10.1093/intqhc/mzm042. 17872937

[nop270166-bib-0035] Ulrich, C. M. , C. H. Rushton , and C. Grady . 2020. “Nurses Confronting the Coronavirus: Challenges Met and Lessons Learned to Date.” Nursing Outlook 68: 1–7. 10.1016/j.outlook.2020.08.018. 33097227 PMC7462465

[nop270166-bib-0036] van der Cingel, M. , and J. Brouwer . 2021. “What Makes a Nurse Today? A Debate on the Nursing Professional Identity and Its Need for Change.” Nursing Philosophy 22, no. 2: e12343. 10.1111/nup.12343. 33450124

[nop270166-bib-0037] Willetts, G. , and D. Clarke . 2014. “Constructing Nurses' Professional Identity Through Social Identity Theory.” International Journal of Nursing Practice 20, no. 2: 164–169. 10.1111/ijn.12108. 24713013

[nop270166-bib-0038] World Medical Association . 2024. “World Medical Association Declaration of Helsinki.” JAMA 353, no. 1: 21972. 10.1001/jama.2024.21972.

[nop270166-bib-0039] Zaghini, F. , V. Biagioli , M. Proietti , S. Badolamenti , J. Fiorini , and A. Sili . 2020. “The Role of Occupational Stress in the Association Between Emotional Labor and Burnout in Nurses: A Cross‐Sectional Study.” Applied Nursing Research 54: 151277. 32650898 10.1016/j.apnr.2020.151277

[nop270166-bib-0040] Zaghini, F. , J. Fiorini , L. Livigni , G. Carrabs , and A. Sili . 2021. “A Mixed Methods Study of an Organization's Approach to the COVID‐19 Health Care Crisis.” Nursing Outlook 69, no. 5: 793–804. 10.1016/j.outlook.2021.05.008. 34176670 PMC8114768

[nop270166-bib-0041] Zapf, D. , and M. Holz . 2006. “On the Positive and Negative Effects of Emotion Work in Organizations.” European Journal of Work and Organizational Psychology 15, no. 1: 1–28. 10.1080/13594320500412199.

[nop270166-bib-0042] Zerbini, G. , A. Ebigbo , P. Reicherts , M. Kunz , and H. Messman . 2020. “Psychosocial Burden of Healthcare Professionals in Times of COVID‐19—A Survey Conducted at the University Hospital Augsburg.” German Medical Science: GMS e‐Journal 18: Doc05. 10.3205/000281. 32595421 PMC7314868

